# Supplementary material for: Predicting visual function by interpreting a neuronal wiring diagram
Source: Nature. 2024 Oct 2;634(8032):113–23. doi: 10.1038/s41586-024-07953-5 (PMC11446822; doi:10.1038/s41586-024-07953-5)
Supplement: Supplementary file 6 — CRF and ERF predictions for individual TmY4 and TmY9 cells. Analogous to Supplementary Data 3, but for TmY target types. Shown are the top four monosynaptic pathways, the strongest pathway passing through each of the top ten intermediary types (ranking from Extended Data Fig. 7), and the trisynaptic pathway Tm1–TmY–Dm3–TmY (see the section entitled Prediction of spatial normalization). [file 41586_2024_7953_MOESM6_ESM.zip › DataS4/TmY9q/720575940623319757.pdf]

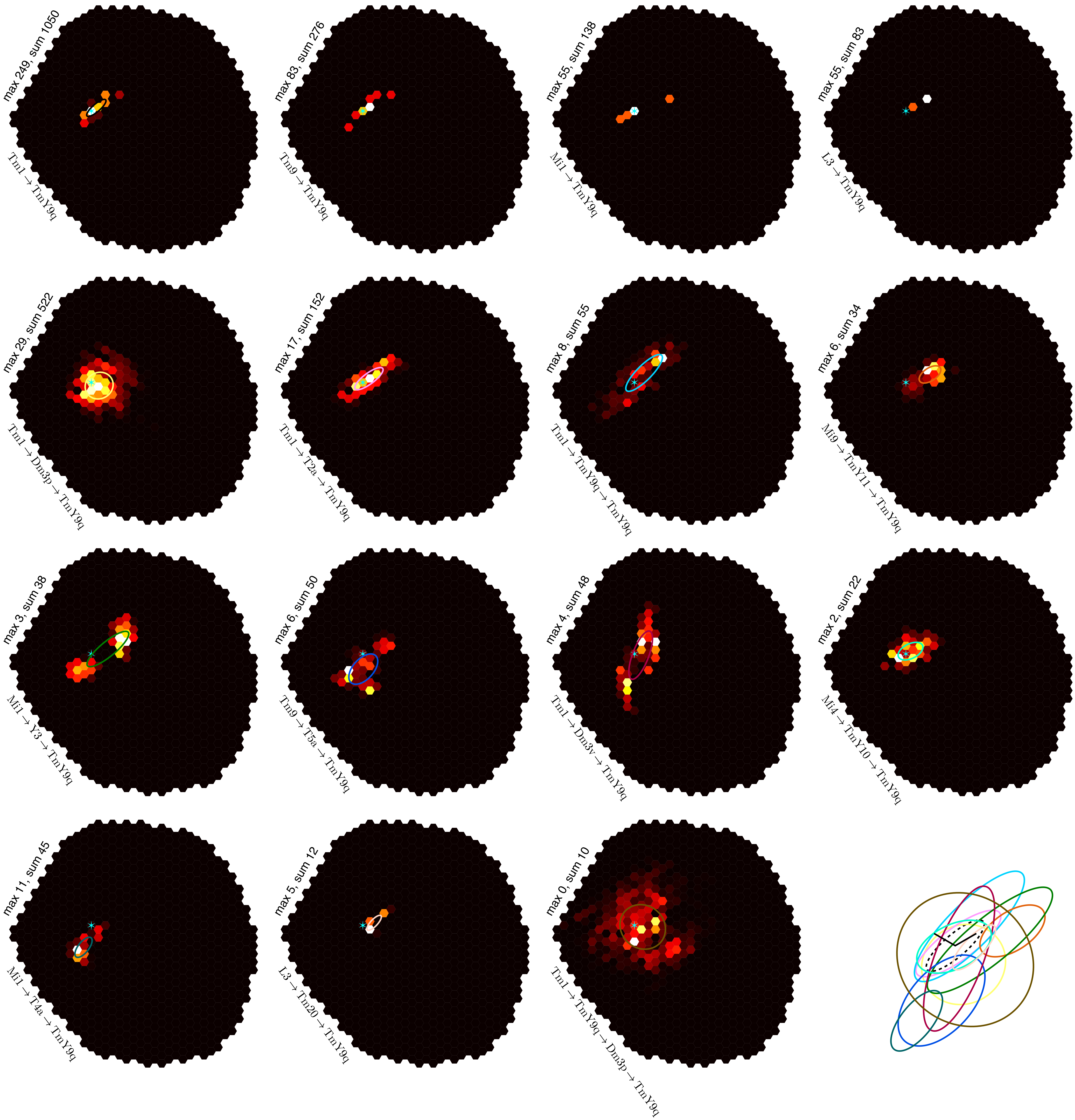
max 249, sum 1050
Tm1 → TmY9q
max 83, sum 276
Tm9 → TmY9q
max 55, sum 138
Mi1 → TmY9q
max 55, sum 83
L3 → TmY9q
max 29, sum 522
Tm1 → Dm3p → TmY9q
max 17, sum 152
Tm1 → T2a → TmY9q
max 8, sum 55
Tm1 → TmY9q → TmY9q
max 6, sum 34
Mi9 → TmY11 → TmY9q
max 3, sum 38
Mi1 → Y3 → TmY9q
max 6, sum 50
Tm9 → T5a → TmY9q
max 4, sum 48
Tm1 → Dm3v → TmY9q
max 2, sum 22
Mi4 → TmY10 → TmY9q
max 11, sum 45
Mi1 → T4a → TmY9q
max 5, sum 12
L3 → Tm20 → TmY9q
max 0, sum 10
Tm1 → TmY9q → Dm3p → TmY9q
